# Interleukin-37: The Effect of Anti-Inflammatory Response in Human Coronary Artery Endothelial Cells

**DOI:** 10.1155/2019/2650590

**Published:** 2019-01-08

**Authors:** Xianfeng Yan, Bin Xie, Guihai Wu, Jing Hu, Di Wang, Xiangna Cai, Jilin Li

**Affiliations:** ^1^Department of Cardiology, First Affiliated Hospital of Shantou University Medical College, Shantou, Guangdong 515041, China; ^2^Department of Cardiology, Jiangxi Provincial People's Hospital, Nanchang, Jiangxi 330006, China; ^3^Department of Dermatovenereology, Second Affiliated Hospital of Shantou University Medical College, Shantou, Guangdong 515041, China; ^4^Department of Plastic Surgery, First Affiliated Hospital of Shantou University Medical College, Shantou, Guangdong 515041, China

## Abstract

Interleukin-37 (IL-37) is unique in the IL-1 family since it broadly suppresses innate immunity and elevates in humans with inflammatory and autoimmune diseases. IL-37 shows definite groups and transcripts for human IL37 gene, but it is still not completely understood the effect and mechanisms of inflammatory response in endothelial cells. It is well accepted that endothelial dysfunction caused by inflammation is a key initiating event in atherosclerotic plaque formation, which leads to the occurrence and development of the cardiovascular adverse events in clinical since the inflammatory responses of endothelial cells could induce and enhance the deposition of extensive lipid and the formation of atherosclerotic plaque in the intima. Thus, it is essential to investigate the role and potential mechanisms in endothelial inflammatory response to prevent the formation and development of many cardiovascular diseases including atherosclerosis. So far, the recent studies have revealed that IL-37 is able to inhibit inflammatory response by suppressing the TLR2-NF-*κ*B-ICAM-1 pathway intracellularly in human coronary artery endothelial cells (HCAECs). Further, the role of IL-37 may be related to the IL-18 pathway extracellularly and involved in the adhesion and transmigration of neutrophils in HCAECs.

## 1. Introduction

IL-37 (formerly known as the interleukin-1 family member 7 or IL-1F7 [1, 2]) distinguishes itself from most other anti-inflammatory cytokines by exerting functions that inhibit the responses of a broad spectrum of inflammatory assaults, including proinflammatory cytokines, such as IL-1b, TNF, and TLRs [3–5]. IL-37 has three independent groups and five transcripts [6–8] for the human IL37 gene (IL-37a-e) [9–12]. In vitro, the expression of IL-37 in macrophages or epithelial cells was shown to greatly inhibit constitutive or induced production of several major proinflammatory cytokines, such as IL-1*α*, IL-1*β*, IL-6, MIP-2, and TNF [4, 13]. In vivo, IL-37 protects mice from LPS-induced shock, chemical-induced colitis, and hepatitis [4, 14–16]. Nonetheless, the exact function of IL-37 has remained unapproached.

It is worth noting that transgenic mice expressing the human IL-37 gene have elucidated the anti-inflammatory properties of this cytokine [4]. Transgenic mice expressing human IL-37 (IL37-tg) mice exhibit a prominent protection from myocardial ischemia [4, 14]. IL37-tg mice that suffered from coronary artery ligation had prominently reduced infarct size and improved left ventricular function compared to similarly challenged WT mice. Mice with transgenic expression of IL-37 were protected from lipopolysaccharide- (LPS-) induced shock and exhibited prominent improved endothelial dysfunction after treating with LPS. The previous studies had revealed that the expression of IL-37 in macrophages or epithelial cells almost entirely inhibited production of proinflammatory cytokines, whereas the mass of these cytokines increased with silencing of endogenous IL-37 in human blood cells [17]. And recent research showed that IL-37 is able to offer an anti-inflammatory microenvironment in the aging bone marrow [18]. Thus, IL-37 emerges as a natural inhibitor of inflammatory responses.

As an IL-1 family member, a growing number of the recent reports reveal that IL-37 plays a crucial role in limiting innate inflammation as well as inflammatory action. For example, the recent study illustrates that IL-37 suppresses MyD88-mediated inflammatory responses in human aortic valve interstitial cells [19]. Besides, IL-37 suppresses the inflammatory response to protect cardiac function in old endotoxemic mice [20]. Increased level of IL-37 in patients with Graves' disease (GD) is associated with disease activity, and it plays a protective role against inflammatory effect in GD by inhibiting the production of proinflammatory cytokines [21]. Reducing endogenous IL-37 in human cells reveals that IL-37 limits the production of cytokines induced by IL-1 and Toll-like receptors (TLR) [4] as well as urate crystals [22].

Furthermore, IL-37 plays a critical role in suppressing cardiodepressant cytokines, while it improves LV function in aging mice during endotoxemia through suppression of myocardial production of MCP-1 and cardiodepressant cytokines [23]. Circulating levels of pro-/anti-inflammatory cytokines, including IL-37, may be related to the pathogenesis of atherosclerosis in patients with either acute coronary syndrome or stable coronary artery disease [24]. Our group found that IL-37 may be involved in leukocytic inflammation in acute ST-segment elevation myocardial infarction (STEMI) after PCI [25]. Since the anti-inflammatory response is inhibited in patient with STEMI, the expression of IL-37 is lower in the STEMI than in the healthy [26, 27].

It is worth mentioning that IL-37 exerts inhibitory effect on neuroinflammation in new frontiers. Neuroinflammation induced by the activation of brain nociceptors and neurons is related to mast cells (MCs) which could lead to the secretion of proinflammatory IL-1 family members and TNF. The IL-1 family members and MC-derived TNF which contribute to neuroinflammation by mediating the sensitization of meningeal nociceptors could be inhibited by IL-37 [28]. Similarly, IL-37 also exerts inhibitory effect on inflammation of fibromyalgia. The inflammation of fibromyalgia may derive from the increase in reactivity of central neurons with increased sensitivity localized mainly in the CNS. MCs could release proinflammatory cytokines, chemokines, and chemical mediators when they are involved in the inflammation of fibromyalgia, and the level of TNF induced by MCs elevates in fibromyalgia. At the same time, IL-37 could inhibit the inflammation response of IL-1 family members and TNF in fibromyalgia [29]. Moreover, IL-37 could inhibit macrophage response and its accumulation and reduce the cytokines that mediate inflammatory diseases since IL-37 inhibits innate and adaptive immunities. IL-37 binds IL-18R alpha chain and reduces the production of TNF and inflammatory IL-1 family members induced by MCs since MC activation could contribute to adaptive immunity and inflammation [30]. A recent report manifests that MCs may be activated via the TLR-dependent pathway controlled by MyD88, which could be inhibited by IL-37 [31]. Therefore, at this level, IL-37 is involved and inhibits the MC-mediated adaptive immune and inflammatory responses.

However, the anti-inflammatory effects of IL-37 differ from other anti-inflammatory cytokines such as IL-10 and TGF-beta. On one hand, IL-37 exerts anti-inflammatory responses by suppressing the phosphorylation of NF-*κ*B via MyD88 or N-MyD88 signaling pathway intracellularly. On the other hand, IL-37 also inhibits the formation of functional complex of IL-18R*α*/IL-18/IL-18R*β* by capturing IL-18R*α* on the cell surface extracellularly, to indirectly inhibit the biological activity of IL-18 [32]. In addition, it is also involved in phosphorylation of NF-*κ*B when IL-37 exerts anti-inflammatory responses extracellularly. IL-37 binds to TIR-8 and then inhibits the phosphorylation of NF-*κ*B by regulating signal molecules such as STAT3, p62, and PTEN. So, the forming the functional complex of IL-37/IL-18R*α*/TIR-8 on the cell surface interferes with the function of a typical TIR domain, which hinders the downstream signal transduction of TIRs, including NF-*κ*B [33, 34]. However, the anti-inflammatory function of IL-10 is not only related to the inhibition of NF-*κ*B but also involved in the IL-10-Stat3 signaling. Stat3 is anti-inflammatory for IL-10 while proinflammatory for IL-6 signaling since it induces the expression of Socs3 that regulates various cytokine signaling pathways including IL-6 [35]. It is worth mentioning that the nuclear activity of IL-37 is similar to TGF-*β*. TGF-*β* binds to TGF-*β* receptor II (TGF-*β*RII) triggering the kinase activity of the cytoplasmic domain, which in turn leads to nuclear translocation of Smad molecules and transcription of target genes [36]. However, the presence of the cytokines such as IL-4, 6, 9, 10, and 17 and IFN-*γ* and the interaction of immune cells such as Th17 cells and CD8^+^ T cells are indispensable when TGF-*β* plays a major role under inflammatory conditions [37]. So, this is the biggest difference between TGF and IL-37 in terms of anti-inflammatory.

## 2. IL-37 Is a “Dual Function” Anti-Inflammation Cytokine

Interestingly, IL-37 is regarded as a “dual function” anti-inflammation cytokine [38]. On one hand, the translocation of IL-37 to the nucleus requires Smad3 when it takes a biological effect [39]. Caspase-1 processing is required for maturation of the intracellular IL-37 precursor and for the translocation of the cytokine to the nucleus [40]. Then IL-37 translocates to the nucleus and then comes into being a complex of Smad3 and IL-37, which induces the nuclear activity of IL-37 [13]. IL-37 interacted intracellularly with Smad3 and IL-37-expressing cells, and transgenic mice exhibited less cytokine inhibition when endogenous Smad3 was depleted. IL-37 is stored intracellularly and rapidly released when the cell encounters an inflammatory assault, which may combat inflammation in a time-efficient manner without recruiting de novo synthesis. Thus, the intracellular way enables IL-37 to mediate anti-inflammatory actions effectively and briskly. On the other hand, the IL-37 precursor is exported from the cell into the extracellular space to take a series of biological effects. IL-37 is biologically active when released into the extracellular space since neutralizing antibodies did not reverse the anti-inflammatory properties in IL-37tg mice [40]. Thus, in this regard, IL-37 is termed as a “dual function” cytokine since it plays the biological role both intracellularly and extracellularly, similar to IL-1*α* [41] and IL-33 [42].

However, how does IL-37 limit inflammation? The previous studies have shown that IL-37 is a new anti-inflammatory cytokine of the IL-1 family, which regulates the inflammation of specific organ or tissue by independent receptor such as IL-1*α* and IL-33 [43]. Since IL-37 has been demonstrated in a variety of cell types from different species, including in humans and rodents [4, 44], it is supposed to have an anti-inflammatory function by the downregulation of proinflammatory cytokines. The recent studies revealed the potential anti-inflammatory mechanism of IL-37 both extracellularly and intracellularly.

**Figure 1:**
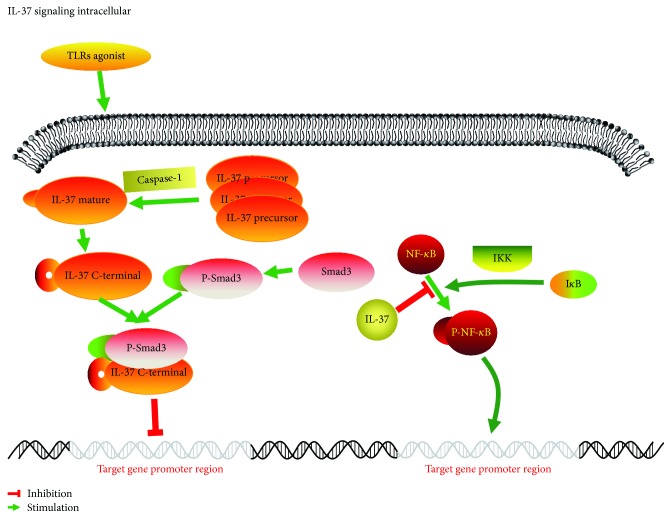
IL-37 is a “dual function” anti-inflammation cytokine. IL-37 exerts anti-inflammatory responses beginning with the nuclear activity intracellularly. IL-37 can recruit Smad3 in the cytoplasm and then comes into being a complex of Smad3 and IL-37, which induces the nuclear activity of IL-37. Then the complex translocates into the nucleus to suppress TLR-induced proinflammatory cytokine. Meanwhile, NF-*κ*B may be phosphorylated by IKK in the cytoplasm and then exposures to the nuclear to regulate the transcription of proinflammatory cytokine, such as ICAM-1, VCAM-1, MCP-1, TNF-*α*, and IL-6. But the phosphorylation of NF-*κ*B can be inhibited by IL-37, which in turn decreases the production of proinflammatory cytokine.

IL-37 is able to inhibit the expression of ICAM-1 following the activation of TLR2 or TLR4 in HCAECs, which is related to the NF-*κ*B pathway [45]. As mentioned above, IL-37 is a “dual function” cytokine that exerts anti-inflammatory effects both intracellularly and extracellularly. NF-*κ*B is prevalent in the cells of the organism, and it is trapped in the cytoplasm when the cells are not stimulated by the inhibitory protein I*κ*B [46]. I*κ*B is degraded by the kinase IKK (I*κ*B kinase) when activated by external stimuli, resulting in the phosphorylation of dissociative NF-*κ*B in the cytoplasm and the exposure to the nuclear localization signal [47], and then transferred to the nucleus and combines with the promoter region of the target gene in the nucleus, which can regulate the transcription of inflammatory cytokine, such as ICAM-1, VCAM-1, MCP-1, IL-6, IL-8, and TNF-*α* [48–50]. IL-37 contains a caspase-1 cleavage site that is present in the cytoplasm in the form of a precursor [13], which is activated by the stimulation of inflammation in parallel with cleaving the IL-37 precursor into a mature one. Translocation of the IL-37 and Smad3 complex to the nucleus inhibits signal transduction proteins, resulting in the suppression of TLR-induced proinflammatory cytokine and the dendritic cells (DCs) [40, 51] (Figure 1).

**Figure 2:**
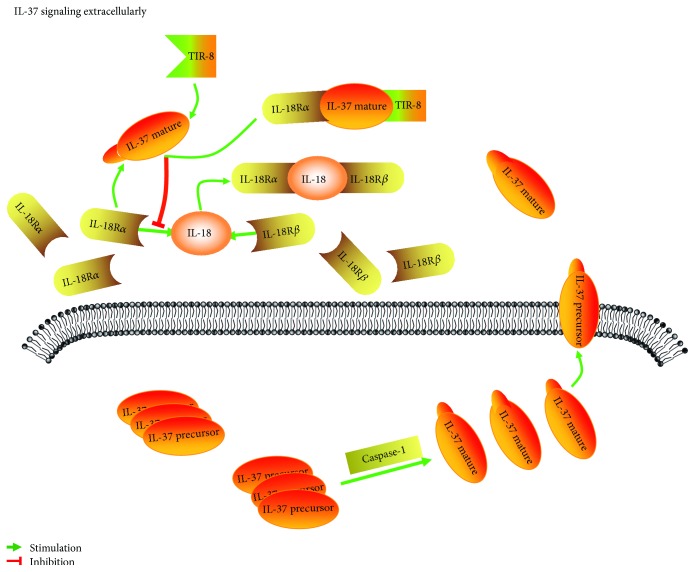
IL-37 is a “dual function” anti-inflammation cytokine. IL-37 exerts a biological role extracellularly. The IL-37 precursor is exported from the cell into the extracellular space to take a series of biological effects. IL-18 binds to IL-18R*α* at the cell surface and recruits the IL-18R*β* chain to form a functional complex. IL-37 is combined with IL-18R*α* side chain to prevent the formation of IL-18 functional complex.

Extracellular effect of IL-37 relies on IL-18BP (interleukin 18-binding protein, IL-18BP) [52, 53]. Early studies already revealed that IL-18 binds to its receptor IL-18R*α* (IL-18 receptor-*α*, IL-18R*α*) at the cell surface and recruits the IL-18R*β* (IL-18 receptor-*β*, IL-18R*β*) chain to form a functional complex [32, 54, 55], and it combines with TIR to regulate the transcription of proinflammatory cytokines with the activation of NF-*κ*B. It can prevent the formation of IL-18 functional complex while the IL-37 is combined with the IL-18R alpha side chain (Figure 2). IL-18BP, a natural inhibitor of IL-18 in humans [56], may bind endogenous IL-37 to upregulate the suppression to IL-18 [52, 57]. However, the affinity of IL-18 with IL-18R*α* and IL-18BP is higher than that of IL-37, so IL-37 cannot inhibit the effect of IL-18 effectively [54]. The recent studies have found that the combination of IL-37 and IL-18R*α* can recruit TIR-8 to form a functional complex in the cell surface. TIR-8, unlike traditional TIR structures, is able to interfere with the activation of traditional TIR domains [33]. Thus, TIR-8 plays a negative role in the activation of downstream signaling pathways induced by the activation of TLR and IL-1R, including NF-*κ*B domain [34]. Besides, another study has found that knockout of TIR-8 in human and mouse cells increases responsiveness to LPS stimulation and results in the production of a large number of proinflammatory cytokines [58]. IL-37 binds to TIR-8 and then inhibits the phosphorylation of NF-*κ*B by regulating signal molecules such as STAT3, p62, and PTEN [5].

## 3. IL-37 Exerts Anti-Inflammatory Responses in HCAECS

The IL-37 protein has been shown to be expressed by many tissues and cells of human, such as blood monocytes [59], tissue macrophages, and plasma cells. IL-37 is constitutively induced by TGF-*β*, TLR agonists [4], beta-defensin-3 in human keratinocytes [60], or the type II epithelial cell of the human lung [61]. But the abundance of IL-37 transcripts is low in human blood monocytes and dendritic cells (DCs) [62]. Concentrations of IL-37 in the circulation of healthy humans are low (<100 pg/mL) but rise with diseases such as rheumatoid arthritis [63], lupus [64], and preeclampsia [65].

IL-37 exerts anti-inflammatory responses in many types of cells, such as peripheral blood mononuclear cells (PBMCs) [21, 64, 66, 67], renal tubular epithelial cells [68], and HCAECs [45]. A recent study indicated that IL-37 could suppress the production of proinflammatory cytokines in monosodium urate (MSU) crystal-induced inflammatory response in PBMCs [22]. Analogously, an early study suggested that a decreased IL-37 expression in Behçet disease (BD) patients was associated with an increased inflammatory response. Another study found that the increased proinflammatory cytokine production from asthma-induced sputum mononuclear cells was abrogated by the addition of rIL-37 [69]. Interestingly, some researchers found that human renal tubular epithelial cells expressed the IL-18 contraregulatory protein IL-37 as an endogenous control mechanism to reduce inflammation [68]. What is more, an increasing number of the recent studies have shown that IL-37 exerts anti-inflammatory responses in HCAECs.

Moreover, the recombinant forms of IL-37 have been shown to be active in vivo in wild-type (WT) mice that suffered from various models of inflammation. The recent studies have shown that IL-37 could inhibit myocardial inflammation, which protects against cardiac dysfunction during endotoxemia in old mice. LV function was improved since myocardial inflammatory responses to endotoxemia in old mice were suppressed by IL-37, as the result of the attenuation of NF-*κ*B activation and MCP-1 production following LPS stimulation in cardiac microvascular endothelial cells from IL-37tg mice [20]. Similarly, recombinant IL-37 treatment in WT mice also provides protection in models of myocardial infarction [70]. Silencing of IL-37 in human blood monocytes results in a 2- to 3-fold increase in LPS and IL-1b-induced cytokines, suggesting that endogenous IL-37 serves as a natural brake of inflammation [6].

### 3.1. The Inflammation Response Is Related to the TLR2/4-NF-*κ*B Pathway

The effect and mechanism of this anti-inflammatory cytokine have been confirmed after the development of increasing recent studies since IL-37 has an anti-inflammation function in a variety of cell types in humans [4, 44]. The previous studies have shown that IL-37 increases substantially in peripheral blood of patients with acute myocardial infarction [71]. What is more, IL-37 is found to ameliorate the inflammatory process in psoriasis by inhibiting the production of proinflammatory cytokine [72]. A recent study reveals that IL-37 may improve the cardiac function in myocardial infarction (MI) mice via inhibition of the inflammatory NF-*κ*B signaling pathway [73]. NF-*κ*B, as a kind of an important nuclear transcription factor, not only plays an important role in inflammation but also relates to myocardial cell apoptosis and myocardial remodeling process after MI [74–76]. In this study, they proved that the inhibition of the NF-*κ*B signaling pathway can improve cardiac function after MI and prognosis since NF-*κ*B can regulate many proinflammatory cytokine transcriptions such as TNF*α*, IL-6, and monocyte chemotactic protein (MCP-1), which not only cause myocardial cell hypertrophy and apoptosis but also affect the myocardial systolic function leading to ventricular remodeling occurrence and heart failure [77–79].

On the TLR2/4-NF-*κ*B signaling pathway, several previous studies have made some demonstrations about its potential mechanism. An early study that explores the role of S100A1 in hypoxia-induced inflammatory response in cardiomyocytes has demonstrated that S100A1 treatment significantly enhanced IL-37 protein or mRNA level, which in turn could attenuate ROS and phospho-p65 NF-*κ*B levels. Thus, the researchers finally considered that S100A1 could regulate the inflammatory response in H9C2 cells via TLR4/ROS/NF-*κ*B pathway [80–82]. A recent study also further confirmed that IL-37 exerts anti-inflammatory effects via TLR2/4-NF-*κ*B signaling pathway. The study revealed that IL-37 reduced the production of these inflammatory mediators induced by TLR4 whereas knockdown of IL-37 enhanced the induction of these mediators by TLR4. Further, IL-37 not only suppressed inflammatory mediator production induced by the MyD88-dependent TLR2 but also inhibited NF-*κ*B activation induced by TLR2 or TLR4. So, they finally demonstrated that IL-37 suppressed MyD88-mediated responses to reduce inflammatory mediator production via TLR2/4-NF-*κ*B pathway in human aortic valve interstitial cells (AVICs) [19, 83]. Analogously, a recent study has revealed that IL-37 could suppress the inflammatory mediator production in human AVICs via this pathway [84]. The study aimed at making an investigation of the proinflammatory signaling pathway responsible for the anti-inflammatory mechanisms in AVICs suggested that IL-37 suppress AVIC osteogenic responses through inhibition of NF-*κ*B since it had augmented inflammatory response to TLR2/4 agonists [74, 85, 86].

### 3.2. IL-37 Exerts Anti-Inflammation Response via TLR2-NF-*κ*B-ICAM-1 Pathway in HCAECs

TLR2/4 activation could increase the IL-37 expression, while the expression of ICAM-1 could be decreased in the mouse coronary microvascular endothelial cells isolated from the IL-37-tg, which may be in parallel with the reduction of NF-*κ*B phosphorylation [20]. And our group found that ICAM-1 and NF-*κ*B expression prominently reduced the overexpression of recombinant IL-37b, while this effect is not indicated even abrogated prominently when IL-37 mRNA is silent [45]. Thus, the effect and mechanism of IL-37 on ICAM-1 expression may be due to the decrease of NF-*κ*B.

Some scholars already found that TLR2 activation made the upregulated expression of the inflammatory cytokine ICAM-1 and NF-*κ*B in the inchoate study of HCAECs [87]. They demonstrated that diabetic HCAECs have enhanced inflammatory responses to TLR2 and TLR4 agonists with increased expression of ICAM-1, IL-6, and IL-8. The hyperinflammatory phenotype of diabetic HCAECs is characterized by augmented NF-*κ*B activation in response to TLR2/4 agonists in the absence of altered cellular TLR2/4 levels. Furthermore, in the recent study, we firstly affirmed that the expression of IL-37 in HCAECs could be regulated when treated with IL-37 transfection plasmid or silent mRNA for 24 h. IL-37 was able to decrease ICAM-1 and NF-*κ*B expression mediated by TLR2 activation in HCAECs, while ICAM-1 and NF-*κ*B expression were increased when IL-37 was knocked out. So, the reduction effect of IL-37 on ICAM-1 expression may be due to the inhibition of activation of NF-*κ*B. And the phosphorylation of NF-*κ*B in intranuclear leads to TLR activation. Translocation of the activated NF-*κ*B into the nucleus to bind to the NF-*κ*B-specific DNA-binding sites, resulting in the regulation of the target genes transcription, such as ICAM-1. Besides, IL-18 pathway may be another potential mechanism on the decreasing effect of IL-37 on ICAM-1. IL-18 is suppressed by IL-37 through enhancing the effect of IL-18BP, which in turn decreases the expression of ICAM-1 [88]. So far, the exact effect and mechanism of the IL-18 pathway are still not well demonstrated and need a further study to make it clear.

**Figure 3:**
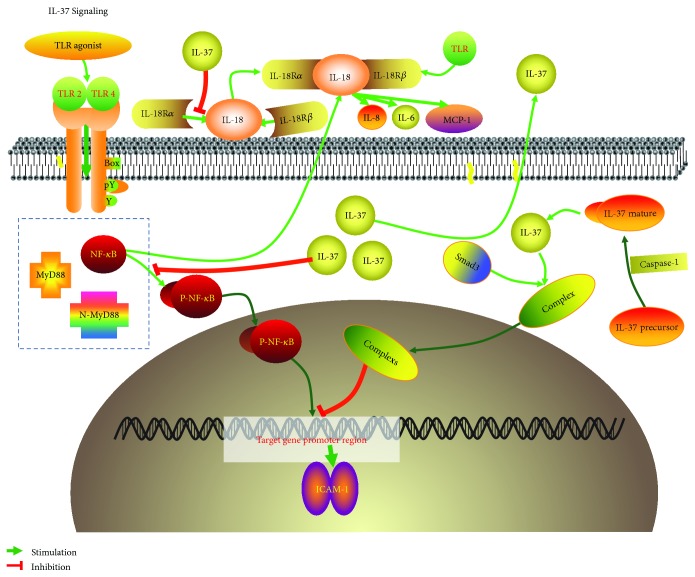
IL-37 signaling pathway induced by TLR2/4 extracellularly and intracellularly. The translocation of the activated NF-*κ*B induced by TLR2/4 into the nucleus for binding to NF-*κ*B-specific DNA-binding sites, resulting in the regulation of the transcription of target genes, such as ICAM-1. On one hand, IL-37 can inhibit the TLR2/4-NF-*κ*B signaling pathway by suppressing the phosphorylation of NF-*κ*B or binding to Smad3 to inhibit transcription in the nucleus. On the other hand, IL-37 can combine with IL-18R*α* side chain to prevent the formation of IL-18 functional complex to suppress the production of proinflammatory cytokine extracellularly.

In this study, we finally confirmed that IL-37 which exerts its effect on the ICAM-1 level by reduction of NF-*κ*B phosphorylation has anti-inflammation function upon TLR2 activation in HCAECs. But the inhibition of IL-18 may be another possibility of inhibited effect of IL-37 on ICAM-1 independent on NF-*κ*B phosphorylation. In conclusion, the suppression of the TLR2-NF-*κ*B-ICAM-1 pathway in HCAECs can prominently reduce the development of inflammatory reaction while TLR2 activation can increase ICAM-1 expression [87] (Figure 3).

Correspondingly, an experiment in a mouse myocardial ischemia/reperfusion (I/R) injury model has further demonstrated that IL-37 exerts its anti-inflammatory effect via TLR-4/NF-*κ*B inflammation pathway. In this study, they found that NF-*κ*B activation was suppressed significantly in the IL-37-treated group compared with the untreated group, which is consistent with the results of the reduced production of inflammatory cytokines [89]. Moreover, they also found that TLR-4 expression was inhibited significantly in IL-37-treated I/R mice compared with untreated mice since the inhibition of the inflammation response was due to the decreased expression of TLR-4, an upstream signaling of NF-*κ*B [89]. Finally, they also confirmed that IL-37 could suppress the expression of proinflammatory cytokines such as TNF-*α*, IL-6, and IL-1*β* by downregulating TLR-4/NF-*κ*B signaling [90].

### 3.3. IL-37 Involves in the Adhesion and Transmigration of Neutrophils in HCAECs

Moreover, IL-37 is also associated with the adhesion and transmigration of neutrophils in HCAECs. Neutrophils are not only involved in endothelial cell-mediated inflammatory response but also tended to release inflammatory cytokines expressing proteins and other cytokines to induce the adhesion of other polymorphonuclear cells, so as to further promote inflammation and atherosclerosis progression [91–94]. The endothelial inflammatory response includes the expression of cell adhesion molecules such as intercellular adhesion molecule-1 (ICAM-1) that allow circulating neutrophils to adhere to sites of endothelial injury [95–97], which is involved in the pathogenesis and development of atherosclerosis [98–100]. The recent studies have found that TLR [101, 102] activation can induce endothelial inflammatory responses and then followed by the adhesion and transmigration of the neutrophils [103–106], while this transmigration can be reduced when TLRs are inhibited [107–110]. So some scholars insist that IL-37 can decrease the ICAM-1 expression in HCAECs stimulated with TLR2/4 activation, followed by the reduction of adhesion and transmigration of neutrophils [111–113]. Heretofore, the previous studies have demonstrated that the protective effects of IL-37 are associated with reduced IKK phosphorylation, NF-*κ*B intranuclear translocation, MCP-1 production, and mononuclear cell accumulation in the ischemic myocardium of the mouse coronary microvascular endothelial cells isolated from the IL-37-tg mice. And the similar results have been proved in human aortic valve interstitial cells stimulated with the TLR2/4 agonist. Furthermore, the current research is focus on researching the effect of the adhesion and transmigration of neutrophils in HCAECs to make a further confirmation of this possible mechanism.

Correspondingly, a study which revealed that IL-37 inhibits neutrophil recruitment through modulating chemokine expression in vivo and migration ability in vitro in a mouse myocardial ischemia/reperfusion (I/R) injury model has further demonstrated our hypothesis mentioned above. In this study, they found that IL-37-treated mice showed less infiltration of inflammatory cells, which, as a result of IL-37 treatment, decreased the infiltration of neutrophils significantly after I/R injury compared with the control mice. LIX, as a potent chemoattractant for neutrophil infiltration [114] was obviously suppressed by IL-37, to inhibit the migration of neutrophils in a concentration-dependent manner, which indicated that the *in vivo* IL-37-mediated inhibition of neutrophils within the ischemic myocardium in I/R mice may be due to their suppressed migration potential and the decreased expression of chemokines [90].

## 4. Prospects

A large number of studies have confirmed that coronary atherosclerosis (AS) begins as a chronic inflammatory disorder characterized by a condition within the arterial wall in which the accumulation of cells, cholesterol, and extracellular matrix causes the hardening of the arterial wall [115, 116]. In the process of atherosclerosis, macrophage and foam cells accumulated under the blood vessel endothelium cells, especially in the plaque [117]. Proinflammatory and anti-inflammatory cytokines as well as inflammatory cells play a pivotal role in the development of inflammation. Once the balance of proinflammatory and anti-inflammatory cytokines is broke, it can promote the plaque progress and the plaque becomes unstable, which results in clinical acute cardiovascular events [118]. Studies have revealed that the concentration of IL-37 increases significantly in the foam or macrophage cells of the atherosclerotic plaque, suggesting that IL-37 released from the atherosclerotic lesion to the blood may play a protective role in the development of atherosclerosis [119–121]. Predictably, from this perspective, IL-37 may be involved in atherosclerosis-related diseases since it is expressed in the foam-like cells of atherosclerotic coronary and carotid artery plaques. IL-37 induced in an inflammatory context may be associated with the development of atherosclerosis while it plays a vital role in the development of inflammatory reaction through inhibiting the production of inflammatory cytokine mentioned above [74, 122].

From this review, we hypothesize that IL-37 may provide a new therapeutic approach to antagonize or delay the formation of atherosclerosis, to prevent or delay the formation and development of coronary atherosclerotic heart disease or acute coronary syndromes. Thus, it is worth for us to make a further study on the anti-inflammatory mechanism and clinical application of IL-37. However, the formation and progression of the disease are a complex and multifactorial process. Therefore, study and cognition of a single factor for disease intervention and treatment unlikely achieve the desired result. We should focus on more than one factor at the cellular and molecular level to do more study of the disease, in order to make a more comprehensive and systematic cognition to its pathophysiology.

Many cytokines or chemokines are involved in the process of anti-inflammatory reaction in HCAECs. For example, a recent study has illustrated that TNF*α* and cigarette smoke extract synergise to induce expression of the transcriptional regulator activating transcription factor 3 (ATF3), which is able to decrease inflammatory gene expression independently of the activation of NF-*κ*B in HCAECs. So, the modulation of ATF3 expression may represent a novel approach to modulate proinflammatory gene expression and open new therapeutic avenues to treat proinflammatory diseases [123]. Besides, the upregulation of microRNA-138/130a could alleviate HCAEC injury and inflammatory response [124, 125]. What is more, amino acids may exhibit anti-inflammatory effects during endothelial inflammation in HCAECs [126].

In short, the intervention for a variety of factors including IL-37 in the inflammatory response of endothelial cells will provide an innovative way of therapeutic potential and strategy in order to give a more comprehensive and systematic treatment of inflammatory diseases such as atherosclerosis.

## 5. Conclusions

Currently, a growing bodies of research have revealed the view that IL-37 plays a vital role to inhibit various inflammation responses especially in the formation of atherosclerosis in cardiovascular disease. The TLR2-NF-*κ*B-ICAM-1 pathway in HCAECs which could be suppressed by IL-37 expression may be associated with the development of inflammatory reaction. A large amount of data and experiments have confirmed that IL-37 emerges as a possible new therapeutic approach to suppress inflammatory diseases. So how to enhance the effect of IL-37 may be the next study point in the future.

## Abbreviations

HCAECs: Human coronary artery endothelial cells
TLR: Toll-like receptors
NF-*κ*B: Nuclear transcription factor-*κ*B
ICAM-1: Intercellular adhesion molecule-1
VCAM-1: Vascular cell adhesion molecule-1
MCP-1: Monocyte chemotactic protein-1
MIP-2: Macrophage inflammatory protein-2
PBMCs: Peripheral blood mononuclear cells
AVICs: Aortic valve interstitial cells
TGF-*β*: Transforming growth factor *β*
Th17 cells: T helper 17 cells
Mast cells: MCs
DCs: Dendritic cells
IL-37: Interleukin-37
IL-1R: Interleukin-1 receptor
IL-1F7: Interleukin-1 family 7
LPS: Lipopolysaccharide
IL37-tg: Transgenic mice expressing human interleukin-37
IL-18BP: Interleukin 18-binding protein
IL-18R*α*, *β*: Interleukin-18 receptor-*α*, *β*
MyD88: Myeloid differentiation factor 88
I*κ*B: Inhibitor protein of NF-*κ*B
IKK: Inhibitor of NF-*κ*B kinase
TIR-8: Toll/IL-1 receptor-related-8
TNF-*α*: Tumor necrosis factor-*α*
ATF3: Activating transcription factor 3
ROS: Reactive oxygen species
STEMI: ST-segment elevation myocardial infarction
PCI: Percutaneous coronary intervention
AS: Atherosclerosis.
